# Supplementary material for: Efficacy of Emu Oil Transfersomes for Local Transdermal Delivery of 4-OH Tamoxifen in the Treatment of Breast Cancer
Source: Pharmaceutics. 2020 Aug 25;12(9):807. doi: 10.3390/pharmaceutics12090807 (PMC7558379; doi:10.3390/pharmaceutics12090807)
Supplement: Supplementary file 1 [file pharmaceutics-12-00807-s001.pdf]

pharmaceutics

Supplementary Materials: Efficacy of Emu Oil Transfersomes for Local Transdermal Delivery of 4-OH Tamoxifen in the Treatment of Breast Cancer.

Usha Sundralingam, Srikumar Chakravarth, Ammu K. Radhakrishnan, Saravanan Muniyandy and Uma D. Palanisamy

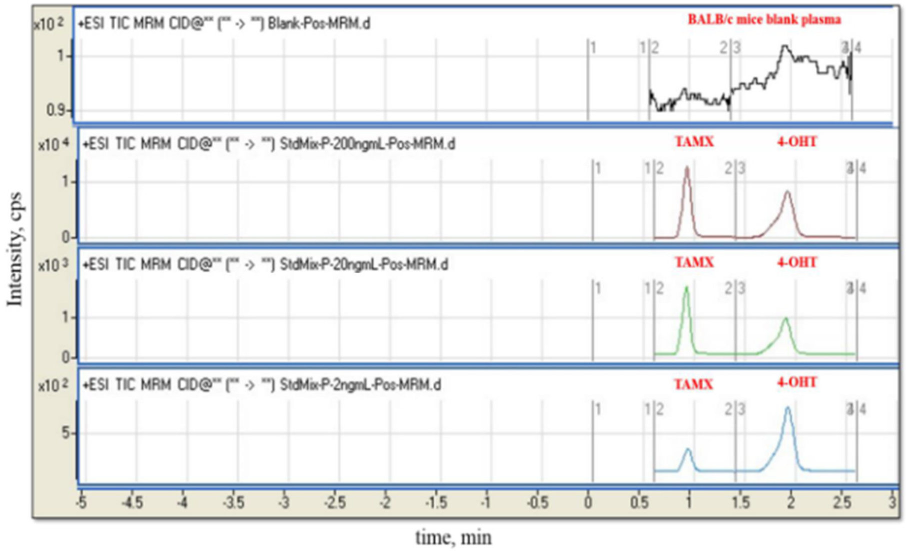

Figure S1. Typical MRM chromatograms of TAMX and 4-OHT in BALB/c mice plasma at three different concentrations (200, 20 and 2 ng/ml) of TAMX and 4-OHT spiked in the mice plasma compared against blank plasma.
